# Patient perspectives of asthma treatment and belief barriers to implementing new guidelines: a qualitative analysis of an online forum

**DOI:** 10.1038/s41533-026-00483-9

**Published:** 2026-05-15

**Authors:** Zoe Moon, Hannah Truscott, Grainne d’ Ancona, Anna De Simoni, Louise Fleming, Mark L. Levy, Aziz Sheikh, Robert Horne

**Affiliations:** 1 Centre for Behavioural Medicine, University College London, London, UK; 2 University of Leeds, Leeds, UK; 3 Guy’s and St Thomas’ NHS Foundation Trust, London, UK; 4 Queen Mary University London, London, UK; 5 Royal Brompton Hospital, Imperial College, London, London, UK; 6 Semi-Retired General Practitioner, London, UK; 7 Nuffield Department of Primary Care Health Sciences, University of Oxford, Oxford, UK

**Keywords:** Diseases, Health care, Medical research

## Abstract

For the last 40 years, the first line of asthma treatment has been short-acting beta2 agonists (SABA) used as symptom relief alongside daily inhaled corticosteroids (ICS) as preventers. New guidelines recommend replacing SABA relievers with ICS-based relievers (ICS-formoterol) because of clear risks from excess and sole use of SABA. This study aimed to explore patient perceptions of SABA, ICS and ICS-formoterol, with a focus on identifying barriers to adherence to new recommended treatments. A qualitative study was carried out investigating UK asthma online community forum posts using keyword searches between July 2021 and March 2025. Inductive thematic analysis was used to analyze the data, informed by the Necessity Concerns Framework. 326 posts were identified across 206 adults with asthma. Thematic analysis highlighted that many people remained attached to their SABA and viewed it as a key element of their treatment. They appeared unconvinced by warnings around SABA over-use and were dissatisfied with the quality of advice from health care professionals (HCPs). In contrast, they raised a range of concerns about ICS and ICS-formoterol, and had reservations about replacing SABA with these treatments. A common objection to replacing SABA was that ICS-formoterol does not provide the same symptom relief. Results highlight a perceived lack of individualized care from HCPs and insufficient explanation and justification of treatment changes. This analysis highlights several potential barriers to adherence to recommended asthma treatment. Strategies to support the implementation of asthma treatment guidelines should include addressing patients’ treatment beliefs and educating clinicians to better communicate treatment changes.

## Introduction

Historically, most people with mild-to-moderate asthma were treated with short acting beta agonists (SABA) used as needed, usually alongside a daily inhaled corticosteroid (ICS) inhaler as maintenance treatment^1^. Evidence however suggests that many people with asthma are overly reliant on SABA, preferring to use this to control symptoms, with low adherence to daily ICS^2–4^. Such an approach is associated with poor asthma control and an increased risk of severe exacerbations and mortality^2^. In 2019, because of the evidence of risks associated with excess SABA and clear benefits of ICS, The Global Initiative for Asthma (GINA) recommended that everyone with asthma should be prescribed ICS-formoterol (ICS combined with a long acting beta_2_ agonist) either as needed for symptom relief (anti-inflammatory reliever, AIR) or regularly as both a Maintenance and Reliever Therapy (MART)^1^.

Similar recommendations were adopted into UK guidance in 2024 based on evidence that the SABA-free pathway reduces the risk of exacerbations by addressing the underlying inflammation^5^ and ensuring that anti-inflammatory treatment is used whenever asthma flares-up^6^.

This represents a significant behavior challenge for healthcare professionals (HCPs) and people with asthma^7–9^. Many people with asthma develop strong emotional connections to their SABA inhaler, perceiving them to be the most effective method for asthma control, attributing this to the rapid relief provided, and that they have “saved them” during critical moments of breathlessness^1,7,10,11^. This, coupled with concerns about the side-effects of steroids^12^, often results in people over-using their SABA and underusing their ICS^3,13^. These beliefs are reinforced by HCP’s continued excess prescription of SABA despite evidence of the risks^14^.

There is a need to understand if these perceptions and behaviors persist in the context of updated treatment recommendations and ongoing approaches to reduce SABA overuse^15,16^. Qualitative research from trials of ICS-formoterol raise potential barriers to its implementation such as low perceived symptom relief^17,18^, but perspectives around ICS-formoterol have not yet been explored outside of a trial setting, or in any depth. Exploring real-world attitudes and barriers related to reducing SABA reliance and increasing the use of ICS treatments is essential for effective guideline implementation and optimal treatment and outcomes.

### Aims and rationale

This study aimed to analyze UK forum posts to explore users’ perceptions of SABA, ICS and ICS-formoterol in asthma care, with a specific focus on understanding barriers to optimal treatment adherence and informing the development of interventions to improve adherence to treatment recommendations. Online discussion forums provide important real-world insights into the lived experiences of individuals^19,20^, and are an alternative to traditional face-to-face focus groups^21,22^.

## Results

Of the posts identified in the search, 323 were judged as relevant and were included in the analysis. A total of 206 users were identified. Most did not state their age, sex or ethnicity. The average number of posts per user was 1.7. Half (49%) of posts analyzed had been added in the past two years (2023–2025).

### Themes

**Fig. 1:**
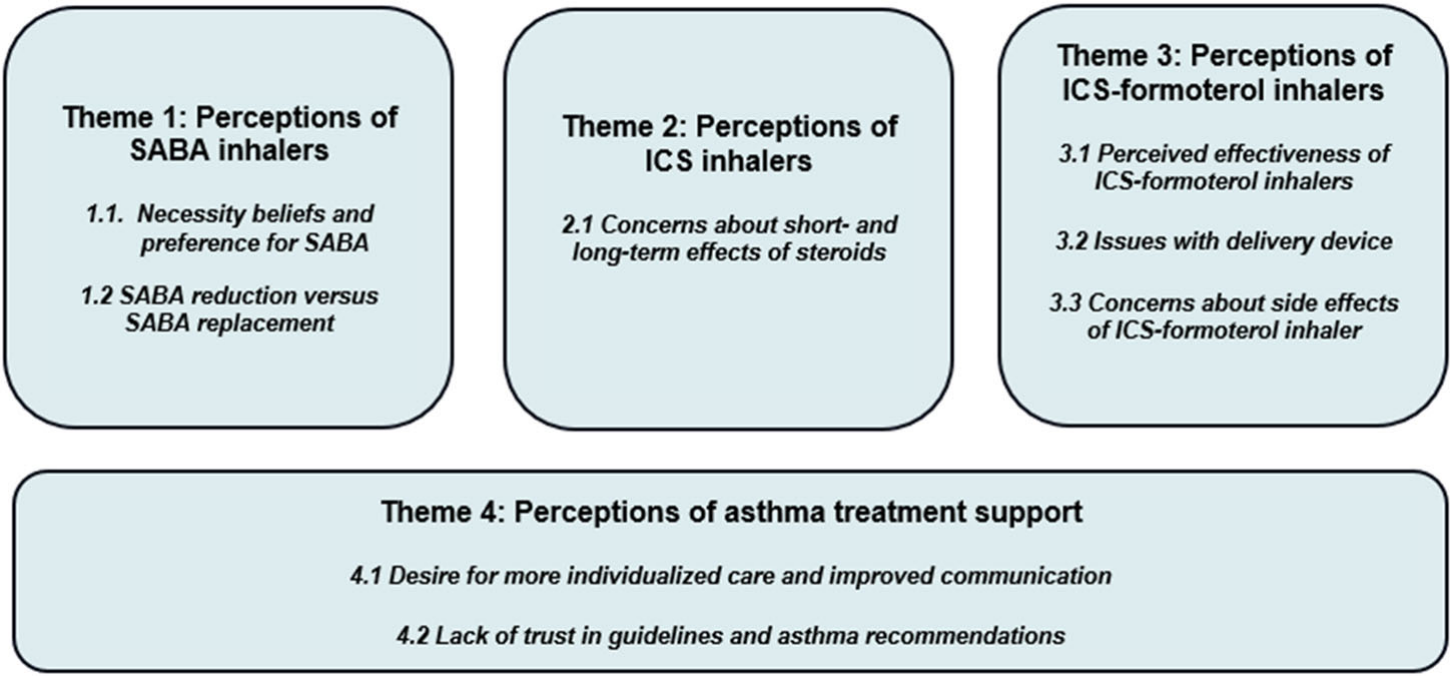
Summary of themes and sub-themes.

Four themes were generated from the analysis: ‘*Perceptions of SABA inhalers’*; *Perceptions of ICS inhalers’*; ‘*Perceptions of ICS-formoterol inhalers’* and ‘*Perceptions of asthma treatment support*’ (see Fig. 1). Additional quotes to support the analysis are shown in Table 1.

**Table 1:** Additional quotes identified.

Subtheme	Quotes
**Theme 1: Perceptions of SABA inhalers**	
1.1 Necessity beliefs and preference for SABA	“*Luckily I still have some of my ‘home’ SABA left but I am worried as the one I carry round with me is running out, so I have a few days to get the prescription before it becomes really worrying.”* (P12, October 2023) *“I have my reliever with me everywhere I go. If I get breathless, I take it*.” (P8, March 2022) *“I’m well controlled now on ICS- vilanterol with SABA but I still take too much SABA out of habit”* (P122, Sept 2023)
1.2 SABA reduction vs SABA replacement	“*Relying on salbutamol, as was common in the past, poses greater risks of severe attacks and means you’ll need courses of oral steroids with more harmful side effects*.” (P17, October 2020) *“Feel free to use the blue inhaler as needed. I used to hesitate. However, waiting until symptoms worsened prolonged my recovery time. Now, I’ve learned that using it at the first sign of chest tightness or puffiness is more effective. This early intervention prevents severe inflammation, reducing the need for repeat doses.”* (P145, November 2023) *“Doctors need to be clear about the differences between ‘needing’ and ‘using’ the reliever… you should not be avoiding use just so you can say you don’t take it more than x times. I’ve seen a few examples of people being told they should just not take it so they can meet a target, and it doesn’t matter how often they actually need to”* (P18, October 2021). *“I’ve been told I can’t have SABA anymore and have to switch to MART. I’m so scared.” (*P193, September 2023)
**Theme 2: Perceptions of ICS inhalers**	
2.1 Concerns about short and long-term effects of steroids	*“I’ve been on the brown inhaler for a few months but I’m thinking of coming off it as my voice is croaky and I have acid reflux. Getting very fed up. I can’t even eat chocolate!!”* (P145, January 2024) *“I weaned myself off my preventer inhaler but now have some symptoms so wondering whether to take it again. I hate taking drugs so I prefer to just use SABA when it’s bad.” (P112, July 2022)* *“I have cut down on my dose of inhaled corticosteroids and take more SABA instead, as it doesn’t give me side effects”* (P115, July 2021)
**Theme 3: Perceptions of ICS-formoterol inhalers**	
3.1 Perceived effectiveness of ICS-formoterol inhalers	*“I take ICS-formoterol, and I used to use my reliever inhaler like 8 times a week. Now, I only need to use it 3 times a week so that’s an improvement.”* (P134, October 2020) *“Last time I ordered salbutamol I got a text saying I shouldn’t need it all. Most of the time my MART reliever works but how are we supposed to cope without the occasional rescue?”* (P126, November 2024)
3.2 Issues with delivery device	“*When I have a flare I can’t get enough breath in or out to actually feel like I was getting the powder from ICS-formoterol into my lungs. Then I have to take so much more to actually feel like it’s getting into my lungs.”* (P185, August 2024) *“I am taking ICS-formoterol as a reliver but often need to use my salbutamol with a spacer as I’m unable to breathe deeply enough to use the powder Symbicort.” (*P180, November 2024)
3.3 Concerns about perceived side effects with ICS-formoterol inhaler	“*ICS-formoterol has formoterol in it, which caused me to have heart palpitations. Despite assurances they would settle, they worsened and it took me a long time to disappear after stopping the inhaler*.” (P128, October 2022) *“I think the LABA component of combination inhalers causes issues for me…I’ve had bad headaches from all the ones I’ve tried.”* (P189, December 2024) *“I’m also thinking about stopping the combination inhalers…no one has been able to explain to me why we are taking these relievers every day when our breathing is fine and is being controlled by the steroid.”* (P22, December 2024)
**Theme 4: Perceptions of asthma treatment support**	
4.1 Desire for more individualized care and improved communication	*“Patronized how to take my inhalers regardless of 50+ years of experience living with asthma”* (P25, September 2022) *“What is the point of an asthma review when the person has no proper understanding about asthma.” (P108, August 2023)* *“In my experiences asthma nurses don’t recognise us as intelligent responsible human beings… they think they know more than us.”* (P16, March 2025) *“The asthma nurse wouldn’t even let me discuss it with her, she just kept telling me to take two doses morning and evening even if I’m feeling fine. It felt like I was being told off by a teacher.”* (P202, September 2024)
4.2 Lack of trust in guidelines and asthma recommendations	*“My doctor has told me that I need to be put on a new preventer inhaler because there are these new rules or guidelines but I told them this is not applicable to me because I don’t have severe asthma.”* (N30; September 2022) *“When will we get to work with medical professionals together to find the best treatment for each individual patient and stop using these tick boxes and quotas?”* (P25; Oct 2022) *“I don’t understand why everyone is being pushed onto MART. What is the logic? I was changed without being asked, and they refused to change me back.”* (P189, December 2024) *“This change from preventer plus blue inhaler to combination inhalers doesn’t sound right to me. They want me to be taking something twice a day and to use the same inhaler if I have an attack. The nurse has not been able to reassure me.”* (P193, September 2023)

#### Theme 1: Perceptions of SABA inhalers

##### Necessity beliefs and preference for SABA

People on the forum reported high necessity beliefs and a strong attachment to their SABA inhalers, making sure they carried them everywhere and always had reserve stock. This attachment to SABA was seen in many people who were also prescribed an alternative ICS-based reliever, who felt that their SABA was the only thing that gave them relief or that they needed to always keep it on hand as a back-up.

*“I’ve never seen steroids do so little! I’m on the MART scheme but maxed out on ICS-formoterol so have been supplementing with a blue… … I know that it’s not sustainable to keep using a blue inhaler like that…but it is the only thing that gives me relief.”* (P95; March, 2023)

*“ICS-formoterol has helped me to reduce my reliance on SABA, but I still carry it everywhere as my safety blanket.” (*P84, November 2024)

Most participants viewed SABA as having none of the side-effects associated with steroids, although some preferred not to use SABA due to the increased palpitations or raised concerns about thin and damaged skin.

##### SABA reduction vs SABA replacement

Many users were aware of the attempts to reduce SABA usage and the messaging around the dangers associated with SABA over-use. Some appeared to understand that relying on SABA was not a effective or safe strategy and could lead to a lack of asthma control. However, most were skeptical about this messaging, interpreting it as a form of ‘*scaremongering’*, ‘guilt-tripping’ or an attempt to reach pre-specified targets:

*“I have been told by an asthma nurse not to take salbutamol [SABA] every time I have shortness of breath. So as long as she ticks her box, I should suffer and struggle for breath or she’ll take it away and I will have to rely on emergency services.”* (P25, Oct 2020)

They felt that healthcare professionals were overly focused on cutting down SABA usage without trying to understand why they may be experiencing symptoms and therefore needing to use their SABA regularly.

*“Instead of worrying about people overdosing, their concern should be that your asthma is very poorly controlled if you need it 4 or more times a day and you should be seeing your GP urgently.” (P18, August,2022)*.

The recommendation to reduce SABA in favor of anti-inflammatory reliever treatment may not be reaching people with asthma in its entirety, as some users appeared to think that doctors had advised them to stop using their SABA without providing any alternative reliever. This was felt to result in people avoiding using their SABA reliever until they were as “*blue as the inhaler*” (P18, August 2022) or “*gulping for air like a fish*” (P12, August 2022). Some were told to stop SABA and switch to the MART regime, which often led to strong feelings of anxiety and frustration.

*“My GP practice is awful. They’ve told me I can’t have my SABA because I’m on MART. Can you believe they are banning reliever inhalers to an asthmatic!” P126, February 2025*

#### Theme 2: Perceptions of ICS inhalers

##### Concerns about short and long-term effects of steroids

Users often attributed side-effects to their ICS, in some cases leading to people discontinuing their treatment.

*“I really want to stop my ICS because of the side-effects. Has anyone tried treating their asthma without steroids?”* (P133, May 2024)

Some appeared to attribute symptoms of worsening asthma to the side-effects of ICS:

“*Steroid inhalers trigger severe reactions for me, leading to asthma attacks. Despite trying several types, each attempt resulted in a harmful response*.” (P72; January 2023)

The perceived harms of ICS were contrasted against the perceived safety of SABA:

*“I’ve been told they are trying to cut down people’s use of salbutamol in preference for inhaled steroids, but I thought salbutamol was much less damaging to the body than steroids”* (P136, October 2020)

#### Theme 3: Perceptions of ICS-formoterol inhalers

##### Perceived effectiveness of ICS-formoterol inhalers

Some users reported positive experiences of their MART regime working and highlighted that it meant they were able to reduce usage of their SABA:

*“I like being on MART as it has helped me to cut down my SABA, which makes it more effective for me, so it’s great all round.”* (P4, Jan 2024)

However, many needed to keep their SABA to hand as they felt their ICS-formoterol reliever did not always help to relieve their symptoms. Some described stopping their ICS-formoterol and switching back to SABA.

*“I was pushed into having ICS-formoterol. There’s a lot of talk about its bronchodilator working for 10* *h, but in reality, SABA is the only thing that truly helps…”* (P159, September 2023)

One user confused a swift onset of relief felt with SABA with a longer lasting effects of a combined ICS-formoterol used as a reliver:

*“The long-acting reliever builds up over a few hours, which is great, but it does not help when I need a speedier relief. I have to use SABA for those moments”* (P158, Nov 2020)

##### Issues with ICS-formoterol inhaler devices

Some felt that they could not use their combined ICS-formoterol dry powder inhaler effectively leading to them using SABA as a reliever instead. This person struggled to breathe deeply enough to feel like the dry powder-based ICS-formoterol relievers were getting into their lungs.

“*I had to resort to using a blue inhaler and spacer because I couldn’t inhale deeply enough to use ICS-formoterol as a reliever….I’ve completely given up on it as reliever and returned to my good old fashioned blue.”* (P68; Female; October 2022)

##### Concerns about perceived side-effects with ICS-formoterol inhaler

Many people suggested that their combination inhaler caused them to experience side-effects, which were attributed to both the formoterol and the ICS. This caused some users to stop taking the treatment, and others to take half the recommended daily dose.

*“I was given ICS-formoterol but stopped taking it after experiencing unexplained sadness, aching in my legs and trembling hands.”* (P154, June 2024)

The majority of users attributed side-effects to the steroid component of the inhaler, but some felt it was the formoterol causing their side-effects. One user prescribed ICS-formoterol as a MART regime was concerned about taking what they perceived to be unnecessary doses of their reliever inhaler.

*“I was wondering why we are given the combined inhaler to take every morning and night regardless of whether we actually need reliever at that time. I thought that taking relievers all the time harms the lungs? it seems like nobody else is any the wiser why we are taking relievers unnecessarily.”* (P22, August 2023)

#### Theme 4: Perceptions of asthma treatment support

##### Desire for more individualized care and improved communication

Across posts, there was a consistent desire for more individualized care. Users argued that as the ones suffering with asthma, they experts in understanding their asthma needs and felt that they were not being listened to. Posts frequently highlighted that people felt HCPs failed to listen to their concerns, resulting in not feeling heard as an individual:

*“I’m fed up with the severity of my asthma being assessed by oral steroid use. They don’t work for me. He’s not following the actual evidence but he doesn’t care, What he says goes.”* (P18; February 2023)

Other users reported they had not been offered an asthma review, “*I have no written plan*” (N127; October 2022), or were told that their doctor had conducted their review without their prior knowledge:

“*My doctor stopped my reliever by doing a medication review which I wasn’t told about. Then the nurse said all asthma patients should have at least one reliever so she added two back on repeat for me*.” (N85; October 2023)

This perceived lack of support led to some people not wanting to seek help.

*“I am almost at the point of cutting off secondary care completely as I see no point in it. The national review of asthma deaths says 45% of asthmatics die without seeking support and I’m beginning to see why*.” *(P151, Nov 2020)*

##### Resistance to guidelines and asthma recommendations

Users described a lack of trust regarding the asthma recommendations (SABA reduction or switching to AIR/MART), and uncertainty as to why they have been changed. Some saw these as cost-saving measures.

*“My asthma nurse said I was managing well on my current regime which does include some SABA, and now they’re saying I don’t need it…is this some kind of cost saving measure? It’ll work if their aim is to kill off half the people with asthma!”* (N12; October 2023)

Others felt their HCPs were more concerned about following recommendations rather than listening to their specific concerns:

*“I’m so fed up with nothing happening. I feel like my health is going backwards and that my doctors do not know what to tell me. It is like they are frightened to not follow the right rules.”* (P39, February 2022)

People resented being switched to MART when they felt they were well controlled and felt they should be able to choose their treatment regime.

## Discussion

This study provides novel insights into perceptions around asthma treatments in the context of new treatment pathways recommending Anti-Inflammatory Reliver (AIR) using ICS-formoterol as a replacement for SABA^5^. Our findings suggest that some forum users continue to use their SABA because they see it as more effective than the ICS-formoterol reliever, while others reported being asked to reduce their SABA use without understanding it should be replaced with an ICS-formoterol based reliever. Interestingly, several users perceived SABA to be relatively harmless and directly contrasted this against the harmful effects of ICS-containing treatments. Some were aware of the risks of SABA over-use but most were skeptical about the warnings and the motives behind them. These perceptions have important implications for implementing new treatment guidelines around decreasing SABA use and increasing the use of ICS-formoterol based treatments.

Our findings are consistent with previous studies showing that people with asthma report strong emotional attachments to and reliance on SABA relievers^7,11^ and support recent trial findings which show that some people with asthma revert back to SABA after being prescribed an ICS-based reliever^18^. However, they add new insights into perceptions of ICS-formoterol and of recent guidelines around reducing SABA prescriptions^8,15^. Our study shows that some users are aware of the warnings around SABA over-use but that many remain unconvinced and prefer to continue using their SABA as previously rather than reducing their usage. Importantly, users felt their doctors should be more concerned about understanding why they were needing to use so much SABA, and addressing this underlying issue, rather than just advising them to cut down their usage. It appears as if some people have been given the advice to reduce their SABA usage, without being given an alternative treatment or without fully understanding the harms of using SABA alone.

Results also support previous literature showing that people with asthma have concerns about using ICS^12^, with some users in this current analysis suggesting that they either had stopped or were considering stopping the treatment due to these concerns. Interestingly, several users directly compared the harm of ICS against SABA, which they incorrectly perceive to be relatively safe^23^. Concerns about side-effects were also reported by users prescribed an ICS-formoterol, with some stopping the treatments due to these side-effects. Taken together, these findings suggest increased education and support is needed to help people understand the need for these new treatments and to overcome concerns about using steroids.

### Strengths and limitations

A strength of using online forum data is that these are less likely than standard qualitative interviews to be affected by self-presentation, reactivity, and recollection biases^24^. Our analysis offers insights into real-world patient perspectives, including people who may not typically participate in research studies, for example from various geographical locations^25^. Evidence suggests people are attracted to the anonymity and convenience of online forums^26^ and feel it is a safe space to engage in open and honest discussions^27^. However, the extracted data were limited to discussed topics and the researcher was unable to probe or clarify. We were unable to extract user characteristics as these were rarely provided by users and we were unable to verify diagnoses or prescriptions. There may be some user bias, as adults in online asthma forums may not represent the wider population, and they may be influenced by what other people have posted. We do not know how widespread these beliefs and concerns are. However, quantitative research suggests that beliefs such as over-reliance on SABA are present in around 50% of people with asthma prescribed SABA. Finally, asthma diagnoses and prescribed treatments are all self-reported and may not be accurate. Due to these inherent limitations, there is now a need to supplement this with additional data such as in-depth qualitative interviews or focus groups.

### Implications

These results highlight the behavioral challenges associated with implementing the new treatment guidelines. Whilst recent initiatives have succeeded in changing SABA prescribing rates^15^, our findings suggest additional support for patients and clinicians may be needed in order for these guidelines to be fully implemented. Results highlight a number of patient perceptions and beliefs which may act as barriers to people receiving optimal treatment, including significant skepticism about replacing SABA with AIR and resistance to SABA reduction, often heightened by poor HCP communication around new guidelines. Identifying and addressing these beliefs is an essential step in changing patient’s behavior to optimize treatment outcomes. This can be done by supplementing consultations with tools like the Reliever Reliance Test^28^, a brief, patient friendly tool to identify and address beliefs associated with SABA over-reliance and over-use, and applying behaviorally intelligent support to address further doubts and concerns^29^. The clinicians’ role in generating these patient misconceptions also needs to be considered, with strategies in place to improve clinician education. Many users in the current study did not think AIR was as effective as their SABA reliever. For some, this was directly attributed to the delivery mechanism, as they found the device too hard to use. Where possible, people with asthma should maintain the same device when switching to AIR. If switching treatments involves a different inhaler, it is likely that additional training will be needed and inhaler technique should be checked^5^.

Users reported being skeptical of treatment recommendations and guidelines which seemed driven by cost-saving or did not feel relevant to them personally. This has been reported in other conditions and highlights a need for improving the way that these treatment changes or recommendations are communicated^30,31^. Future research should investigate effective methods for HCP’s conveying treatment guidelines that consider patients’ pre-existing beliefs and cater to their need for personalized care.

## Conclusions

This online forum revealed a rich source of information regarding perceptions of preventer and reliever inhalers in treating their asthma. Results highlighted several factors which may act as a barrier for people with asthma to reduce their SABA usage or switch to an ICS-formoterol reliever treatment. Awareness of how to identify and modify any misplaced patient perceptions and concerns about these treatments will be key to ensuring optimal patient engagement. Clinicians should consider close follow-up with these patients, to improve the quality of asthma care within the primary care setting.

## Methods

### Design

We undertook a qualitative analytic study on an online discussion forum. We analyzed posts about SABA, ICS and ICS-formoterol from online forums for adults with asthma. HealthUnlocked’s Asthma Community Forum was selected as to date it has over 23,000 members and 25,000 posts. The forum is supported by Asthma+Lung UK, a UK based charity. This study followed the Standards for Reporting Qualitative Research^32^.

### Data collection

Permission from HealthUnlocked was sought prior to identification and use of online forum data for research purposes. To identify relevant forum posts, the following search terms were used to identify posts between October 2019 and March 2025: AIR, ICS, Inhaler, Formoterol, Fostair, LABA, MART, Preventer, Reliever, SABA, Symbicort, Ventolin. Searches were carried out directly in the HealthUnlocked forum search.

Posts were included if they discussed patient perceptions or experiences of the relevant treatments. This included both original posts and replies. Posts written by family members or friends were excluded. Data on usernames, sex, age, and asthma treatment were retrieved where available. Relevant posts were extracted and stored in a Microsoft Excel database. Brand names have been replaced with non-proprietary names.

### Researcher characteristics and reflexivity

In terms of personal values and assumptions of the researchers (HT, ZM), there were no competing interests, although elicitation of emotionally charged stories of asthma may have resulted in an empathetic response, especially when considering concerns relating to asthma-related complications. However, this was acknowledged, and great consideration was taken to set aside any assumptions for the integrity of the study.

### Data analysis

The posts were analyzed using thematic analysis^33^ with an inductive approach informed by the Necessity Concerns Framework, a well validated framework for understanding beliefs about medications^34^. Posts were coded independently by two authors (ZM, HT) and any discrepancies were discussed. Firstly, the researchers familiarized themselves with the data by re-reading the posts. After this, initial codes were generated, highlighting interesting features of the data that appeared meaningful. The codes were collated into potential themes, which were reviewed by all authors, resulting in a ‘thematic map’ of the analysis. Finally, the themes were defined and named, using specific examples from the posts, generating an overall story of the analysis. Coding and themes were revisited and discussed between the researchers until agreement was reached.

### Ethical considerations

Ethics approval for this study was assessed by the University College London’s Research Ethics Committee and was exempt from full review (see Do I need UCL ethical approval? | UCL Research Ethics - UCL – University College London for further information). There is consensus that internet data that are freely and publicly accessible can be used for research without prior ethical approval^35,36^. Details on ethical issues related to analyzing online fora have been described previously as a passive and non-intrusive analysis^37,38^. To protect anonymity, quotes were paraphrased with a focus on ensuring that they still conveyed the original meaning whilst protecting any identifiable information^39,40^.

## Data Availability

Data available upon reasonable request.
